# Improving mental health among people living with HIV: a review of intervention trials in low- and middle-income countries

**DOI:** 10.1017/gmh.2015.17

**Published:** 2015-09-09

**Authors:** K. J. Sikkema, A. C. Dennis, M. H. Watt, K. W. Choi, T. T. Yemeke, J. A. Joska

**Affiliations:** 1 Department of Psychology and Neuroscience, Duke University, Durham, NC, USA; 2 Duke Global Health Institute, Durham, NC, USA; 3 Department of Psychiatry and Mental Health, University of Cape Town, Cape Town, South Africa

**Keywords:** HIV, intervention, low middle income countries, mental health, review, trials

## Abstract

People living with HIV (PLWH) experience greater psychological distress than the general population. Evidence from high-incomes countries suggests that psychological interventions for PLWH can improve mental health symptoms, quality of life, and HIV care engagement. However, little is known about the effectiveness of mental health interventions for PLWH in low- and middle-income countries (LMICs), where the large majority of PLWH reside. This systematized review aims to synthesize findings from mental health intervention trials with PLWH in LMICs to inform the delivery of mental health services in these settings. A systematic search strategy was undertaken to identify peer-reviewed published papers of intervention trials addressing negative psychological states or disorders (e.g. depression, anxiety) among PLWH in LMIC settings. Search results were assessed against pre-established inclusion and exclusion criteria. Data from papers meeting criteria were extracted for synthesis. Twenty-six papers, published between 2000 and 2014, describing 22 unique interventions were identified. Trials were implemented in sub-Saharan Africa (*n* = 13), Asia (*n* = 7), and the Middle East (*n* = 2), and addressed mental health using a variety of approaches, including cognitive-behavioral (*n* = 18), family-level (*n* = 2), and pharmacological (*n* = 2) treatments. Four randomized controlled trials reported significant intervention effects in mental health outcomes, and 11 preliminary studies demonstrated promising findings. Among the limited mental health intervention trials with PLWH in LMICs, few demonstrated efficacy. Mental health interventions for PLWH in LMICs must be further developed and adapted for resource-limited settings to improve effectiveness.

## Introduction

Low- and middle-income countries (LMICs) bear a disproportionate burden of the world's HIV infections, with over 85% of the world's 35 million HIV cases located in LMICs (UN Joint Programme on HIV/AIDS, 2014). Studies across multiple settings have consistently observed that people living with HIV (PLWH) experience greater psychological distress, such as depression and anxiety, as compared with the general population (Bing *et al.* 2001; Ciesla & Roberts, 2001). Evidence from LMICs, though limited, has confirmed high rates of mental disorders and psychological distress among PLWH (Breuer *et al*. 2011; Chibanda *et al.* 2014). Mental disorders in LMICs must be addressed due to their impact on the lives of PLWH, as well as HIV-related outcomes at an individual and population level (Hartzell *et al.* 2008).

Mental disorders and psychological distress not only compromise overall well-being and quality of life among PLWH (Bing *et al.* 2000), but also impact individuals’ ability to engage effectively with HIV care, including their adherence to antiretroviral therapy (ART) (Collins *et al.* 2006; Mayston *et al.* 2012; Uthman *et al.* 2014). The ability to halt disease progression and achieve full viral suppression, which requires proper care engagement, is a primary predictor of HIV infectiousness (Cohen *et al.* 2011). Poor HIV care engagement attributable to unaddressed mental health needs among PLWH may thus hamper proposed efforts to use ‘treatment as prevention’ to curb the spread of the HIV epidemic (Sikkema *et al.* 2010; Gupta *et al.* 2014). Additionally, mental disorders and psychological distress may be associated with HIV risk behaviors such as substance abuse, multiple sexual partners and unprotected sexual intercourse (Crepaz & Marks, 2001), further contributing to the forward transmission of HIV (Senn *et al.* 2010). Taken together, addressing mental health among PLWH appears to be a critical component of HIV treatment and prevention, and should be considered as part of population-level approaches to prevent HIV transmission, particularly in LMICs where the burden of HIV and its associated morbidities is high.

An emerging body of evidence, mostly from high-income settings, suggests that psychological interventions, primarily focused on treating depression and anxiety, can improve the mental health of PLWH (Crepaz *et al.* 2008; Brown & Vanable, 2011; Clucas *et al.* 2011; Harding *et al.* 2011; Sherr *et al.* 2011; Seedat, 2012; Spies *et al.* 2013; Wu & Li, 2013). Among the various treatment modalities, cognitive-behavioral interventions (CBIs) have received the most attention, with skills training and stress management CBIs that include 10 or more sessions demonstrating the greatest improvement in mental health symptoms. These interventions have been found to be equally if not more effective than pharmacological treatments (Clucas *et al.* 2011; Spies *et al.* 2013), though it has been suggested that pharmacological management can be beneficial as an adjunct treatment or combined with psychological approaches (Sherr *et al.* 2011). In addition, CBIs have been found not only to improve mental health symptoms in PLWH, but also to impact HIV-related clinical outcomes, including CD4 counts (Crepaz *et al.* 2008). Furthermore, a limited number of intervention studies have begun to examine the impact of mental health treatment on care engagement and risk behaviors in PLWH. For example, depression treatment has been found to enhance ART adherence (Sin & DiMatteo, 2014), and coping interventions to reduce traumatic stress have been found to decrease substance use (Meade *et al.* 2010) and sexual risk behaviors (Sikkema *et al.* 2008). There is further evidence that psychological interventions can be delivered in community settings, which has the potential for broader reach (Wu & Li, 2013). Intervention studies published subsequent to existing reviews suggest an emerging focus on aging populations (Heckman *et al.* 2013), increased emphasis on treating traumatic stress and post-traumatic stress disorder (PTSD) (Pacella *et al.* 2012; Sikkema *et al.* 2013), and the use of telephone and web-based modalities (Hersch *et al.* 2013; Himelhoch *et al.* 2013; Drozd *et al.* 2014), as well as alternative therapeutic approaches that draw on mindfulness (Gayner *et al.* 2012; Gonzalez-Garcia *et al.* 2014) and expressive writing paradigms (Ironson *et al.* 2013; Carrico *et al.* 2015).

Despite promising intervention efforts and related evidence, most of what has been appraised to date has emerged from high-income countries (HICs). Although previous reviews have included a limited number of studies from LMICs (Crepaz *et al.* 2008; Clucas *et al.* 2011; Harding *et al.* 2011; Sherr *et al.* 2011; Seedat, 2012; Spies *et al.* 2013), thus far, no reviews have focused on interventions that broadly address mental health for PLWH in these unique settings. Despite the dual burdens of HIV and mental disorders in LMICs, and evidence of their synergistic negative effects, strategies to address the mental health needs of PLWH in LMICs have received only limited attention (Mayston *et al.* 2012; Chibanda *et al.* 2014). Mental disorders in these settings often are untreated due to a lack of behavioral and pharmacological treatment opportunities (Kakuma *et al.* 2011; WHO, 2001). This treatment gap has implications for both the well-being of PLWH as well as the effectiveness of national HIV treatment programs (Mayston *et al.* 2012).

There is a need to take stock of existing efforts to respond to mental health distress challenges among PLWH in LMICs, so as to inform the development and scale up of appropriate mental health services in these countries. The goal of this review paper is to synthesize findings from mental health intervention trials for PLWH in LMICs. This information will extend our knowledge of mental health interventions for PLWH beyond those that have been developed and tested in HICs, and will illuminate avenues for future intervention development, testing, and delivery in LMIC settings where there is greatest need.

## Methods

### Inclusion criteria

Studies were included in this review if they met the following criteria:

(1) Described a trial evaluating a mental health intervention (e.g. CBT, coping, cognitive functioning, and pharmacological) among PLWH,
(2) Intervention was implemented in either a LMIC, as defined by the World Bank (The World Bank Group, 2015), or one of the emerging-economy Brazil, Russia, India, China, and South Africa (BRICS) countries (),
(3) Trial assessed at least one negative psychological state or disorder (e.g. depression and anxiety), and
(4) Outcomes were assessed pre- and post-intervention.

### Exclusion criteria

Studies with a heterogeneous (HIV-positive and HIV-negative) sample were excluded if mental health outcomes were not presented separately for the HIV-positive population. Trials with only substance use outcomes were not included in this review. Systematic and non-systematic review articles, as well as studies unavailable in English, qualitative exploratory studies, and studies not published in a peer-reviewed journal were also excluded from this review.

### Search strategy

PubMed, EMBASE, and PsychInfo were searched between 9 and 11 November 2014. Limits to time period were not applied to the search. Standardized search terms and key words related to the constructs of (a) HIV or AIDS, (b) mental health, (c) intervention, and (d) LMIC/BRICS were used in all databases. For example, within PubMed, terms used to capture the construct of mental health included the following: mental health, mental*, depress*, anxi*, trauma*, PTSD, psycholog*, coping, stress, and psychiat*. Search terms for LMIC/BRICS were derived from the World Bank's classification of low-income, lower-middle income, and upper-middle income economies (The World Bank Group, 2015). Conducting the search was a multi-step process. First, separate searches were conducted using HIV/AIDS, mental health, intervention, and LMIC/BRICs search terms, respectively. In the final step, results from these four separate searches were combined using ‘AND’ terms to capture manuscripts that possibly met study inclusion criteria. Where possible, studies with ‘orphan’ in the title were eliminated from the search using the term ‘HIV or AIDS not Orphan’ so as to more efficiently exclude studies focusing on HIV-impacted orphans who were not necessarily HIV-infected. Filters for the above constructs were applied within each database to restrict the search in the following ways:

(1) HIV or AIDS terms were restricted to title only,
(2) Mental health terms were restricted to title or abstract,
(3) Intervention terms were restricted to title only, and
(4) LMIC/BRICS terms were restricted to title, abstract or topic.

### Study selection and data abstraction

A list of all titles and abstracts were considered independently by two researchers, eliminating those studies that did not appear to meet study inclusion criteria; the full study team then reached consensus on articles to be assessed for eligibility. The full text of all relevant articles were then independently reviewed by two researchers to determine inclusion, with >85% agreement between researchers. Discrepancies about the remaining studies were reconciled through discussion with the full study team. Data display matrices were used to extract data from included studies. Data extracted from studies were as follows: author, year, title, date of study, city/country, sample characteristics (age, sex, HIV-status, sample size of intervention and control conditions, mental health inclusion criteria), intervention characteristics (name, level, components, duration, deliverer), study design, evaluation design (control or comparison condition, follow-up, retention), outcome measures, and relevant findings.

## Results

### Search results

**Fig. 1.**
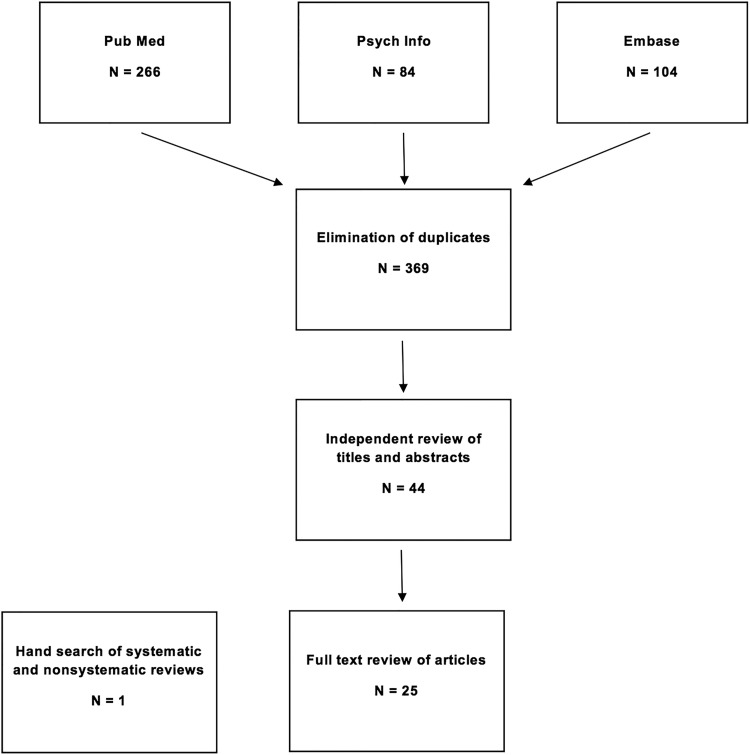
Study selection process.

Initial database searches yielded 454 records, and 369 unique records remained after elimination of duplicates across databases. Screening of titles and abstracts resulted in 44 articles. The full text of these 44 articles was reviewed, which yielded 25 articles (describing 21 unique studies) that met the inclusion criteria for this review (see Fig. 1). One additional article was identified through examination of the reference lists of relevant systematic and non-systematic reviews (Field & Kruger, 2008), for a total of 22 unique intervention studies included in this review. All included studies were published in peer-reviewed journals between 2000 and 2014. The summaries of the 10 randomized control trials (Table 1) and the 12 pilot or feasibility trials (Table 2) are presented separately.

**Table 1.** Randomized controlled trials

Citation(s) and date of study	Location	Sample	Intervention description	Evaluation design^a^	Outcome measures	Relevant findings^b^
Boivin *et al*. (2013) A year-long caregiver training program to improve neurocognition in preschool Ugandan HIV-exposed children Date of study: NR	Uganda	119 child-caregiver dyads: caregivers (>90% HIV + mothers) –Children (aged 2–4 years; all HIV-exposed but uninfected) Intervention (*n* = 59) Control (*n* = 60) No MH inclusion criteria^c^	Name: Mediational intervention for sensitizing caregivers (MISC) Level: individual Components: mediational processes (focusing, exciting, expanding, encouraging, regulating) Duration: MISC sessions delivered biweekly over 1 year, alternating between home and clinic settings Deliverer: MISC trainer trained caregiver	Control: health and nutrition curriculum delivered biweekly over 1 year Follow-up: (p) 12 m Retention: NR	*Caregivers* MH outcomes: depression and anxiety (HSCL-25) Other outcomes: N/A *Children* MH outcomes: –Motor, language, overall cognitive skills (MELS) –Memory (COAT) –Internalizing and externalizing symptoms (CBCL)	*Caregivers (HIV* *+) – No significant effect*^d^ *Children (HIV−) – Significant effect* –Receptive language (12 m) –Expressive language (12 m) –Overall cognitive ability (12 m) –Memory (12 m)
Eller *et al*. (2013) A randomized controlled trial of an HIV/AIDS symptom management manual for depressive symptoms Date of study: 2005–2007	South Africa, Puerto Rico and US	222 HIV + adults with self-reported depression during past week Intervention (*n* = 124) Control (*n* = 98)	Name: HIV/AIDS symptom management manual Level: Individual Components: problem section (describes depressive symptoms), treatment section (describes common treatments from depressive symptoms), and self-care section (describes self-care strategies for depressive symptoms) Duration: self-paced following initial training session Deliverer: self-delivered after 30-min training on use of manual, delivered by research nurse	Control: modified WHO HIV/AIDS nutrition manual. Individual received 30-min session on use of manual. Follow-up: (p) 1, and 2 m Retention: 82% = 1 m 52% = 2 m	MH outcomes: depressive symptoms (CES-D) Other outcomes: self-care behavior (self-care activities checklist)	*No significant effect*
Eloff *et al*. (2014) A randomized clinical trial of intervention to promote resilience in young children of HIV-positive mothers in South Africa Date of study: NR	Pretoria, South Africa	390 mother-child pairs –Mothers (all HIV+) –Child (eldest HIV-child, aged 6–10 years) Intervention (*n* = 199) Control (*n* = 191) No MH inclusion criteria^c^	Name: The Kgolo-Mmogo Project Level: group Components: sessions for mothers focused on issues relating to living with HIV, parent–child interactions and positive parenting behavior. Sessions for children focused on self-esteem and interpersonal skills. Joint sessions focused on parent–child interaction Duration: 24 weekly sessions, each lasting 75 min. First 14 sessions were separate for mothers and children. Last 10 sessions were joint sessions Deliverer: two trained community care workers supervised by a social worker	Control: standard care and information about local resources Follow-up: (p) 6, 12, and 18 m Retention: 74% = 6 m 74% = 12 m 75% = 18 m	*Mothers* MH outcomes: –Depression (CES-D) –Coping (Brief COPE) –Parenting stress (PSI) Other outcomes: –Illness and HIV status disclosure (self-report) –Response to children's negative behaviors (CCNES) *Children* MH outcomes: –Child depression (CDI) –Child anxiety (RCMAS) Other outcomes: –Child behavior (CBCL) –Children's emotional intelligence (The BarOn EQ-I Youth Version) –Children's adaptive functioning (VABS)	*Mothers (HIV+) – No significant effect*^d^ *Children (HIV−) – Significant effect* –Child externalizing behavior (18 m) –Adaptive functioning (18 m) –Daily living skills (18 m)
Kaaya *et al*. (2013) Randomized controlled trial evaluating the effect of an interactive group counseling intervention for HIV positive women on prenatal depression and disclosure of HIV status Date of study: 2001–2004	Dar es Salaam, Tanzania	331 HIV+ pregnant women attending ANC Intervention (*n* = 168) Control (*n* = 163) No MH inclusion criteria	Name: psychosocial group counseling Level: group Components: problem-solving therapy approach to HIV challenges, with sessions on HIV transmission prevention challenges, disclosure and psychosocial support Duration: 6 weekly sessions Deliverer: social worker or psychiatric nurse	Control: standard of care Follow-up: (p) 6 weeks Retention: 57% = 6 weeks	MH outcomes: depression (HSCL-25) Other outcomes: disclosure of HIV status	*No significant effect*
Li *et al*. (2010) Improving the Health and Mental Health of People Living with HIV/AIDS: 12-months Assessment of a behavioral intervention in Thailand --- Li *et al*. (2012) efficacy of an intervention for families living with HIV in Thailand: A randomized controlled trial Date of study: 2007–2008	Thailand	507 HIV+ individuals and 308 HIV-negative family members Intervention (*n* = 260) Control (*n* = 247) No MH inclusion criteria	Name: adaptation of teens and adults learning to communicate (Project TALC, Miller & Rotheram-Borus, 1994) Level: group Components: healthy mind (emotional regulation, positive thinking, HIV disclosure, stress management); healthy body (medication adherence/access to care, prevention of HIV transmission, self-care); Parenting and family relationships (family roles and relationships, parenting while ill, long-term plans with family); social and community integration (community participation and support, employment while ill) Duration: 12–90-min sessions and one preparation session delivered over 13 weeks Deliverer: two trained facilitators	Control: standard of care, including support group for PLWHA and family member Follow-up: 6, 12, 18, 24 m Retention: 98% = 6 m 98% = 12 m 97% = 18 m 89% = 24 m	MH outcomes: general mental health (MOS-HIV subscales for health distress and emotional well-being) Other outcomes: physical and general health (MOS-HIV physical functioning and general health subscales)	*Significant effect* −+Mental health (12 m) +Mental health was only measured at 12 m follow-up
Li *et al*. (2011) A multilevel intervention for HIV-affected families in China: together for empowerment activities (TEA) –-- Li *et al*. (2014) Effect of a family intervention on psychological outcomes of children affected by parental HIV Date of study: 2009–2010	Anhui Province, China	79 families from four villages. All families included at least one HIV+ and one HIV−negative family member. 167 participants intervention (*n* = 80) Control (*n* = 87) No MH inclusion criteria	Name: together for empowerment activities (TEA) Level: multilevel Components: three modules: healthy body and healthy mind, positive family interactions, and Quality of Life Duration: six small group sessions, six home-based family activities and three community events. Intervention activities took about 2.5 months. Deliverer: trained health educators recruited from local agencies	Control: standard of care (educational material and classes on health education, personal hygiene and nutrition) Follow-up: (p) 3 and 6 m Retention: 96% = 3 m 94% = 6 m	*HIV+ Adult* MH outcomes: depression (Zung Self-Rating Depression Scale) Other outcomes: social support (MOS social support survey) –Family functioning (Family functioning scale) *Children* MH outcomes: -self-esteem (RSE) Other outcomes: –perceived parental care (PBI) –Problem behavior (count of list of behaviors related to withdrawal, aggression, and delinquency)	*Adults (HIV+) – Significant effect* –Depressive symptoms (3 and 6 m) –Social support (3 m) –Family functioning (3 m) *Children (HIV-) – No significant effect*^d^
Olley (2006) Improving well-being through psycho-education among voluntary counseling and testing seekers in Nigeria: A controlled outcome study Date of study: NR	Abuja, Nigeria	67 HIV+ individuals recruited from VCT Intervention (*n* = 34) Control (*n* = 33) No MH inclusion criteria	Name: psycho-education level: individual Components: cause and course of HIV/AIDS, its psychosocial impact, and self-management skills Duration: 4 weekly 1-h sessions Deliverer: NR	Control: 4 weekly 1-h sessions of unstructured individual support Follow-up: (p) 4 weeks Retention: 93% = 4 weeks	MH outcomes: depression (BDI); –Generalized anxiety disorder (CCEI) –Coping (Brief COPE) Other outcomes: sexual risk behavior –Self-disclosure intention	*Significant effect* –Depression (4 weeks) –Neurotic disorders (4 weeks) –Safe sex practices (4 weeks) –Self-disclosure of status to partners (4 weeks)
Peltzer *et al*. (2012) Efficacy of a lay health worker led group antiretroviral medication adherence training among non-adherent HIV-positive patients in KwaZulu-Natal, South Africa: Results from a randomized trial Date of study: NR	Kwa Zulu Natal, South Africa	152 HIV+ adults who were new to ARVs and had adherence challenges Intervention (*n* = 76) Control (*n* = 76) No MH inclusion criteria^c^	Name: medication adherence intervention (MAI) Level: group Components: cognitive-behavioral: HIV related knowledge; adherence concerns and patient-specific barriers. Sessions combined medication information with problem-solving skills in an experiential/interactive group format Duration: 3 monthly 1-h sessions. Deliverer: trained lay health worker and adherence counselor	Control: standard of care: (monthly visit to review health status with medical provider, 20 min) Follow-up: (p) 3 and 6 m Retention: 97% = 3 m 97% = 6 m	MH outcomes: depression (BDI-II) Other outcomes: ART adherence (AACTG)	*No significant effect*^d^
Richter *et al*. (2014) Pregnant women living with HIV (WLH) supported at clinics by Peer WLH: a cluster randomized control trial --- Rotheram-Borus *et al*. (2014) A cluster randomized controlled trial evaluating the efficacy of peer mentors to support South African women living with HIV and their infants. e84867 Date of study: 2008–2010	Kwa Zulu Natal, South Africa	1200 HIV+ pregnant women on their first antenatal visit; randomized by clinic recruitment site Intervention (*n* = 544) = 4 clinics: EI +SC: Control (*n* = 656) = 4 clinics: SC No MH inclusion criteria	Name: Masihambisane (‘We Walk Together’) Level: group Components: (1) destigmatizing HIV; (2) adhering to PMTCT tasks; (3) establishing healthy daily routines; (4) infant feeding methods (5) economic support (6) social support; and (7) couples testing, disclosure and condom use Duration: eight, 60–90 min intervention sessions (four antenatal, four postnatal) Deliverer: HIV+ female peer	Control: standard of care (clinical care per the national protocol) Follow-up: (p) 1.5 m post-birth, 6 m post-birth, 12 m post-birth Retention: 70% = 1.5 m post-birth 57% = 6 m post-birth 24% = 12 m post-birth	MH outcomes Depression (GHQ) Other related outcomes: –HIV transmission-related behaviors (self-reported disclosure; request for partner HIV testing) –ARV adherence and engagement in PMTCT protocol Infant health status post-birth	*Significant effect* –Depression symptoms (1.5 m post-birth and 12 m) –Completion of maternal and infant ARV (1.5 m post-birth) –Adherence to all PMTCT tasks through (1.5 m post-birth) –Asking partners to test for HIV (1.5 m post-birth) –Infant health status (all time points)
SeyedAlinaghi *et al*. (2012) Randomized controlled trial of mindfulness-based stress reduction delivered to human immunodeficiency virus-positive patients in Iran: effects on CD4+ T Lymphocyte count and medical and psychological symptoms Date of study: 2008–2010	Tehran, Iran	245 HIV+ adults who had not yet initiated ART Intervention (*n* = 120) Control (*n* = 125) No MH inclusion; Excluded if reported current psychosis or history of PTSD	Name: mindfulness-based stress reduction (MBSR) Level: group Components: mindfulness body scan practices, awareness of body postures using light Hatha yoga, sitting mindfulness meditation, application of MBSR techniques in daily life Duration: 8 weekly sessions Deliverer: psychologist trained in MBSR program	Control: education and support, including educational information and pamphlets about living with HIV/AIDS Follow-up: (p) 8 weeks, 3, 6, 9, and 12 m Retention: NR	MH outcomes: self-reported mental health (SCL-90R) Other Outcomes: CD4+ T lymphocyte count –Physical symptoms (MSCL)	*No significant effect*

a Number of months (m) since baseline; (p) = immediate post assessment.

b No significant effect’ or ‘significant effect’ refer to between-condition effects.

c Indicates that mental health was a secondary outcome.

d No significant difference between conditions; both conditions improved.

NR, not reported; AACTG, Adult AIDS Clinical Trials Group adherence measure; BDI , Beck Depression Inventory; BDI-II, Beck Depression Inventory II; CBCL, Achenbach Child Behavior Checklist; CCBL, Child Behavior Checklist; CCEI, The Crown Crisp Experimental Index; CCNES, Coping with Children's Negative Emotions Scale; CDI, Child Depression Index; CES-D, Center for Epidemiologic Studies-Depression Scale; COAT, Color-Object Association Test; GHQ, General Health Questionnaire; HSCL-25, Hopkins Symptom Checklist; MELS, Mullen Early Learning Scales; MOS-HIV Medical Outcomes Study HIV Health Survey Instrument; MSCL, Medical Symptom Checklist; PBI, Parental Bonding Instrument; RCMAS, Revised Child Manifest Anxiety Scale; RSE, Rosenberg Self-Esteem Scale; SCL-90R, Symptom Checklist-90-Revised; VABS, Vineland Adaptive Behavior Scales.

**Table 2.** Pilot and feasibility studies

Citation(s) and date of study	*Location*	Sample	Intervention description	Study design	Evaluation design	Outcome measures	Relevant findings
Adams *et al*. (2012*a*). Feasibility of nurse-led antidepressant medication management of depression in an HIV clinic in Tanzania. Date of study: NR	Northern Zone of Tanzania	20 HIV+ adults MH inclusion: elevated depression (PHQ-9 ≥10)	Intervention: measurement-based care (MBC) to monitor and treat depression Level: individual. Components: antidepressant medication using MBC treatment algorithm Duration: 12 weeks, with visits at 4, 8, and 12 weeks and optional visits at 2, 6, and 10 weeks to monitor side effects Deliverer: nurse and clinical officer	Pre-experimental, one-group pre-post design O X O	Comparison: none Follow-up: post Retention: 85% = post	*HIV+ Participants* MH outcomes: depression (PHQ-9) Other outcomes: –Antidepressant and antiretroviral therapy adherence (AACTG)	−Depression decreased from baseline to post −100% adherence to antidepressants when prescribed
Bhana *et al*. (2014). The VUKA family program: piloting a family-based psychosocial intervention to promote health and mental health among HIV infected early adolescents in South Africa. Date of study: NR	KwaZulu-Natal, South Africa	65 families with an HIV+ child aged between 10 and 14 years Intervention (*n* = 33) Control (*n* = 32) No MH inclusion criteria	Intervention: VUKA family program Level: group Components: session topics included: AIDS related loss and bereavement, stigma and discrimination, social support and adherence among other topics Duration: Six sessions over 3 m (two sessions per month) Deliverer: lay counselors supervised by one masters level psychologist	Randomized, pre-post, wait list control group pilot design R O X O R O O X	Control: wait list control (*n* = 32); received VUKA intervention after the study ended (3 m later) Follow-up: post Retention: 91% = post	*HIV+ Youth* MH outcomes: child depression (CDI) –Youth mental health (SDQ) Other outcomes: –Adherence to ART (PACTG) –HIV treatment knowledge –Self-concept (TSCS) Caregiver: –HIV/AIDS stigma –Youth and caregiver communication and comfort	*HIV+ Youth* –Improvements in ART adherence and in HIV treatment knowledge –No findings on depression reported *Caregivers (HIV status NR)* –Less stigma and greater comfort in communicating with the children
Chan *et al*. (2005). Cognitive-behavioral group program for Chinese heterosexual HIV-infected men in Hong Kong. Date of study: NR	Hong Kong, China	16 adult males with symptomatic HIV Intervention (*n* = 8) Control (*n* = 8) No MH inclusion criteria	Intervention: cognitive-behavioral program (CBP) intervention Level: group Components: cognitive restructuring and behavior change strategies; psychoeducation on stress; stress management techniques Duration: 7 weekly sessions Deliverer: clinical psychologist and trainee	Randomized, pre-post, wait list control design R O X O R O O X	Control: wait list control Follow-up: post Retention: 81% = post	*HIV+ Participants* MH outcomes:depression (CES-D) Other outcomes: health-related quality of life (SF-36)	−Reductions in depression and distress
Field and Kruger (2008). The effect of an art psychotherapy intervention on levels of depression and health locus of control orientations experienced by black women living with HIV. Date of study: 2008	Soshanguve, South Africa	18 HIV+ women attending an HIV support group Intervention (*n* = 9) Comparison (*n* = 9) MH inclusion: Elevated depression (BDI-II > 14)	Intervention: art psychotherapy Level: group Components: doll making workshop as expressive therapy and used in cognitive feedback Duration: One 6 h workshop Deliverer: researcher trained in art therapy	Experimental, pre-test, post-test and post-post-test design O X O O O O O	Comparison: timed matched entertainment workshop. Follow-up: post, 2 weeks Retention: 100% = post 100% = 2 weeks	*HIV+ Participants* MH outcomes: depression (BDI-II) Other outcomes: health locus of control (MHLCS)	−Improvements in depression and health locus of control
Jirapaet (2000). Effects of an empowerment program on coping, quality of life, and the maternal role adaptation of Thai HIV-infected mothers. Date of study: 1998	Bangkok, Thailand	94 HIV+ mothers Intervention (*n* = 46) Comparison (*n* = 48) No MH inclusion criteria^a^	Intervention: empowerment program (EP) Level: group Components: participatory action; solutions to live with HIV as manageable illness. Duration: 6 weekly sessions. Deliverer: researchers acting as facilitators	Pre-post, non-equivalent control quasi-experimental design O X O O O	Comparison: standard of care Follow-up: post Retention: NR	*HIV+ Participants* MH outcomes: coping ability (JCS) Other outcomes: maternal role adaptation (MCQ) –Quality of life (SPQL)	−Improvements in coping, quality of life and maternal role adaptation
Molassiotis *et al*. (2002). A pilot study of the effects of cognitive-behavioral group therapy and peer support/counseling in decreasing psychologic distress and improving quality of life in Chinese patients with symptomatic HIV disease. Date of study: NR	Hong Kong, China	46 adults with symptomatic HIV Intervention 1 (CBT) (*n* = 10) Intervention 2 (PSC) (*n* = 10) Control (*n* = 26) No MH inclusion criteria	Intervention: (1) Cognitive-behavioral group therapy (CBT) (2) Peer support/counseling group therapy (PSC) Level: group Components: (1) CBT: cognitive restructuring, behavior change strategies, assertive skills, relaxation training, coping, supportive relationships and disclosure (2) PSC: CBT topics without training in behavior change and cognitive problem solving skills Duration: CBT and PSC: 12 sessions, delivered over 3 m Deliverer: Qualified nurse supervised by mental health nurse and psychologist.	Randomized, pre-post pilot trial design R O X_1_ O O R O X_2_ O O R O O O	Control: crisis intervention and individual counseling as needed Follow-up: post, 3 m Retention: NR = post 78% = 3 m	*HIV+ Participants* MH outcomes: negative mood states (POMS) Other outcomes: quality of life (WHOQOL-BREF-HK) –Uncertainty in illness (MUIS)	−Improvements in overall mood states (CBT and PSC) –Improvements in depression, tension-anxiety, anger-hostility and quality of life (CBT)
Mundell *et al*. (2011). The impact of structured support groups for pregnant South African women recently diagnosed HIV positive. Date of study: 2005–2006	Pretoria, South Africa	361 HIV+ pregnant women Intervention (*n* = 144) Comparison (*n* = 217) No MH inclusion criteria	Intervention: structured psychosocial support group intervention Level: group Components: sessions covered relational issues, coping, stigma and stress management Duration: ten sessions, weekly Deliverer: master's level psychology students and HIV+ women from the community	−Quasi experimental, Pre- post design with convenience comparison group O X O O O O O	Comparison: women who declined to join support groups Follow-up: post, 8 m Retention: 77% = post and/or 8 m	*HIV+ Participants* MH outcomes: depression (CES-D) –Coping (Brief COPE) Other outcomes: disclosure, self-esteem (RSE) -Social support (MSSI)	−No difference between groups for depression or social support –Improvements in coping and self esteem
Nyamathi *et al*. (2012). Impact of an ASHA intervention on depressive symptoms among rural women living with AIDS in India: comparison of the Asha Life and Usual Care Program. --- Nyamathi *et al*. (2013). Impact of Asha intervention on stigma among rural Indian women with AIDS. Date of study: 2009–2011	Andhra Pradesh, India	68 HIV+ women Intervention (*n* = 34) Control (*n* = 34) No MH inclusion criteria	Intervention: ASHA-Life Level: individual Components: coping with HIV/AIDS, ART knowledge, parenting, coping, nutrition, and life skills. Weekly visits by trained lay-village women (ASHA) to assist in adherence and care. Duration: six sessions and weekly visits Deliverer: health care providers, trained lay-village woman (ASHA)	−Pilot prospective study using cluster randomization R O X O R O O	Control: usual care: matched sessions in length and time to intervention Follow-up: 6 m from baseline Retention: 100% = 6 m	*HIV+ Participants* MH outcomes: sepression (CES-D) Other outcomes: avoidant coping –Stigma –Knowledge about HIV (HIV-KQ)	−Decrease in depressive symptoms
Pence *et al*. (2014). Feasibility, safety, acceptability, and preliminary efficacy of measurement-based care depression treatment for HIV patients in Bamenda, Cameroon. Date of study: 2011	Bamenda, Cameroon	55 HIV+ patients MH inclusion: major depressive disorder (PHQ-9 ≥10 and physician assessment)	Intervention: measurement-based Care (MBC) to monitor and treat depression Level: individual Components: prescription of anti-depressant medication and monitoring of depression measures to adjust dose Duration: 12 weeks Deliverer: non-physician depression care manager	Pre Experimental, one-group pre-post design O X O	Comparison: none Follow-up: post Retention: 100% = post	*HIV+ Participants* MH outcomes: depression (PHQ-9) Other outcomes: N/A	−87% of participants achieved remission of depression
Petersen *et al*. (2014). A group-based counseling intervention for depression comorbid with HIV/AIDS using a task shifting approach in South Africa: a randomized controlled pilot study. Date of study: 2012–2013	KwaZulu Natal province, South Africa	76 HIV+ ART clinic patients Intervention (*n* = 41) Control (*n* = 35) MH inclusion: major depressive disorder (SRQ-20 >8, confirmed with SCID-II)	Intervention: group-based IPT Level: group Components: problem management and cognitive behavioral techniques to address triggers of depression (poverty, grief, interpersonal conflicts, and externalized stigma, exacerbating factors, social isolation and intrusive negative thoughts, internalized stigma) Duration: 8 weekly sessions Deliverer: lay HIV counsellors	Randomized, pre-post, control design R O X O R O O	Control: standard of care, including counseling Follow-up: post Retention: 45% = post	*HIV+ Participants* MH outcomes: depression (PHQ-9, HSCL-25) Other outcomes: sSocial support (MSPSS)	−Reductions in depression
Ravaei *et al*. (2013). Effectiveness of cognitive behavioral and spiritual trainings on improving mental health of HIV positive drug addicts. Date of study: 2009	Terhan, Iran	30 HIV+ drug using males Intervention (*n* = 15) Control (*n* = 15) No MH inclusion criteria	Intervention: cognitive behavioral and spiritual training Level: group Components: stress and anger management, endurance, self-awareness, cognitive errors and negative beliefs. Focus on drug effects, HIV prevention and reinforcing spiritual beliefs and their effects on mental health. Duration: 8 weekly sessions Deliverer: NR	Pre-post with control group R O X O R O O	Control: no treatment Follow-up: post Retention: 100% = post	*HIV+ Participants* MH outcomes: mental health (MOS-HIV) Other outcomes: –N/A	−Improvement in mental health
Yu *et al*. (2014). A pilot theory-based intervention to improve resilience, psychosocial well-being, and quality of life among people living with HIV in rural China. Date of study: 2008–2009	Rural China	75 HIV+ adults, infected via blood/plasma donations 36 HIV-negative community members No MH inclusion criteria	Intervention: intervention to improve resilience, psychosocial well-being, and QOL Level: group (groups included both HIV+ and HIV− participants) Components: improving resilience (self-worth, emotional control, optimism, social support, and empathy toward vulnerable people) Duration: 8 biweekly sessions conducted over 4 m Deliverer: family planning staff	Single-arm open evaluative study using a pre-and post-intervention study design O X O	Comparison: none Follow-up: post, 3 m Retention: 88.3% = post 76.6% = 3 m	*HIV+ Participants* MH outcomes: resilience (CD-RISC); depression, anxiety, and stress (DASS) -Social support (MOS)	−Higher resilience, social support, and quality of life –Reductions in depression, anxiety, and stress

a Indicates that mental health was a secondary outcome.

MH, Mental Health; NR = not reported; AACTG, Adult AIDS Clinical Trial Groups Adherence Measure; BDI-II, Beck Depression Inventory; Brief COPE, abbreviated version of the COPE inventory; CBP, Cognitive Behavioral Program; CBT, Cognitive Behavioral Therapy; CES-D, Center for Epidemiologic Studies Depression Scale; CDI, Child Depression Inventory; CD-RISC, Connor-Davidson Resilience Scale; DASS, Depression, Anxiety, and Stress Scale; HIV-KQ, HIV Knowledge Questionnaire; HSCL-25, Hopkins Symptom Checklist; JCS, Jalowiec Coping Scale; MBC, Measurement Based Care; MCQ, Maternal Caregiving Questionnaire; MHLCS, Multidimensional Health Locus of Control Scale; MOS, Medical Outcomes Study; MOS-HIV, Medical Outcomes Study HIV Health Survey Instrument; MSSI, Multidimensional Social Support Inventory; MSPSS, Multidimensional Scale of Perceived Social Support; MUIS, Mishel Uncertainity in Illness Scale; PACTG, Pediatric AIDS Clinical Trial Groups Adherence Measure; PHQ-9, Patient Health Questionnaire-9; POMS, Profile of Mood States; PSC, Peer support/counseling group therapy; QOL, Quality of Life; RSE, Rosenberg Self-Esteem Scale; SCID-II, Structured Clinical Interview for DSM Disorders; SDQ, Strengths and Difficulties Questionnaire; SF-36, Medical Outcomes Study Short-Form 36; SPQL, Perceived Life Quality Index; SRQ-20, Self-Reporting Questionnaire; TSCS, Tennessee Self-Concept Scale; WHOQOL-BREF-HK, World Health Organization Quality of Life scale.

### Randomized controlled trials (RCTs)

#### Study location

RCTs were conducted in Uganda (Boivin *et al.* 2013), South Africa (Peltzer *et al.* 2012; Eller *et al.* 2013; Eloff *et al.* 2014; Richter *et al*. 2014; Rotheram-Borus *et al*. 2014), Tanzania (Kaaya *et al*. 2013), Nigeria (Olley 2006), China (Li *et al.* 2011, 2014), Thailand (Li *et al.* 2010, 2012), and Iran (Seyed Alinaghi *et al.* 2012).

#### Participants

A total of 2893 participants were reported across all trials, of which 1664 individuals participated in the experimental intervention condition. A median sample size of 233.5 at baseline (range 67–1200) was reported across all trials. All studies included HIV-positive participants, yet study samples were diverse. Four RCTs were designed for implementation within families (Li *et al.* 2010, 2011, 2012, 2014; Boivin *et al.* 2013; Eloff *et al.* 2014); no studies included only child or adolescent participants. Of the six remaining RCTs, four were designed for implementation with HIV-positive men and women (Olley, 2006; Peltzer *et al.* 2012; SeyedAlinaghi *et al.* 2012; Eller *et al.* 2013), and two were for women only (Kaaya *et al.* 2013; Richter *et al*. 2014; Rotheram-Borus *et al*. 2014). Only one study had a specified mental health inclusion criterion, depressive symptoms (Eller *et al.* 2013). None of the RCTs utilized a mental disorder as inclusion criteria.

#### Study design

One RCT (Li *et al.* 2011, 2014) randomized by cluster; all other RCTs randomized individual participants to the intervention or control condition. In all studies, outcomes were assessed post-intervention (median retention rate reported of 87.5%, range 57–98%), with most trials also reporting longer-term follow-up assessments between 2 and 18 months (median retention rate reported final follow-up of 82%, range 24–97%). (Note: outcomes in Tables 1 and 2 correspond to post-intervention measurements, unless otherwise noted).

#### Intervention content and delivery method

The interventions reported in these studies had diverse characteristics. Most interventions were designed to be implemented with groups (Li *et al.* 2010, 2012, Peltzer *et al.* 2012; SeyedAlinaghi *et al.* 2012; Kaaya *et al.* 2013; Eloff *et al.* 2014; Richter *et al*. 2014; Rotheram-Borus *et al*. 2014), and one group-based intervention was multilevel, including family and community components (Li *et al.* 2011, 2014). Individual-based interventions (1–26 sessions) sought to address neurocognitive (Boivin *et al.* 2013), psychological (Eller *et al.* 2013), and behavioral (Olley, 2006) outcomes through approaches utilizing psycho-education (Olley, 2006; Eller *et al.* 2013) or focusing on meditational processes for cognitive outcomes (Boivin *et al.* 2013). Group interventions (3–24 sessions) primarily addressed psychological (Li *et al.* 2010, 2011, 2012, 2014), behavioral (Li *et al.* 2010, 2012, Peltzer *et al.* 2012; Kaaya *et al.* 2013; Eloff *et al.* 2014; Richter *et al*. 2014; Rotheram-Borus *et al*. 2014), and biological (SeyedAlinaghi *et al.* 2012) outcomes through approaches utilizing mindfulness meditation (SeyedAlinaghi *et al.* 2012), skills training, problem solving, or cognitive behavioral approaches (Li *et al.* 2010, 2011, 2012, 2014; Peltzer *et al.* 2012; Kaaya *et al.* 2013; Eloff *et al.* 2014; Richter *et al*. 2014; Rotheram-Borus *et al*. 2014). Interventions were delivered by a wide range of professionals and non-specialists. Only one study (Boivin *et al.* 2013) noted the cultural appropriateness of the tested intervention.

#### Control/comparison groups

For most studies, control participants received the standard of care (Li *et al.* 2010, 2011, 2012, 2014; Peltzer *et al.* 2012; SeyedAlinaghi *et al.* 2012; Kaaya *et al.* 2013; Eloff *et al.* 2014; Richter *et al*. 2014; Rotheram-Borus *et al*. 2014); three studies (Olley, 2006; Boivin *et al.* 2013; Eller *et al.* 2013) utilized an attention-matched control intervention.

#### Outcome measures

Mental health outcomes were reported in all studies (Table 1); all primary mental health outcomes were measured using standardized psychological symptom scales. None of the studies reported including a diagnosis of mental disorder. Outcomes related to depression were reported in all but two studies (Li *et al.* 2010, 2012, SeyedAlinaghi *et al.* 2012). In the latter, composite mental health outcome measures were utilized. See Table 1 for mental health and related psychological assessments utilized across studies. Six studies (Olley, 2006; Li *et al.* 2010, 2012; SeyedAlinaghi *et al.* 2012; Boivin *et al.* 2013; Kaaya *et al.* 2013; Eloff *et al.* 2014) noted attention to cultural adaptation of measures or validation of measures in the country in which the study took place.

#### Intervention effects

Four of the 10 studies reported a significant between-condition intervention effect for PLWH (Olley, 2006; Li *et al.* 2010, 2011, 2012, 2014; Richter *et al*. 2014; Rotheram-Borus *et al*. 2014). Further, Boivin *et al*. (2013) and Eloff *et al*. (2014), both family studies with HIV-infected caregivers and HIV-uninfected children, found significant effects in the child sample but non-significant effects in the HIV-infected adult caregivers; notably, these studies were designed to improve neurocognitive (Boivin *et al.* 2013) and resiliency (Eloff *et al.* 2014) outcomes in children. Four studies (Peltzer *et al.* 2012; SeyedAlinaghi *et al.* 2012; Eller *et al.* 2013; Kaaya *et al.* 2013) resulted in positive but non-significant intervention effects on mental health outcomes.

### Pilot, feasibility, and quasi-experimental studies

In addition to the 10 RCT studies described in detail above, 12 additional preliminary studies were identified that provide future directions for mental health intervention with PLWH in LMIC settings. These studies, details shown in Table 2, were either described by the authors as pilot trials (many with RCT methods) or were not conducted using an RCT design.

#### Study location

Pilot, feasibility, and quasi-experimental studies were conducted in, Tanzania (Adams *et al.* 2012*a*), South Africa (Field & Kruger, 2008; Mundell *et al.* 2011; Bhana *et al.* 2014; Petersen *et al.* 2014), Cameroon (Pence *et al.* 2014), China (Molassiotis *et al.* 2002; Chan *et al.* 2005; Yu *et al.* 2014), Thailand (Jirapaet, 2000), India (Nyamathi *et al.* 2012, 2013), and Iran (Ravaei *et al.* 2013).

#### Participants

A total of 924 HIV-infected individuals participated across all studies. A median baseline sample size of 60 (range 16–361) was reported across all trials. The median retention rate reported post-intervention was 88.3% (range 45–100%). Study samples were diverse, and included drug-addicted males (Ravaei *et al.* 2013), women only (Nyamathi *et al.* 2012, 2013), pregnant women or mothers (Jirapaet, 2000; Mundell *et al.* 2011), children (Bhana *et al.* 2014), patients screened for depression (Field & Kruger, 2008; Adams *et al.* 2012*a*; Pence *et al.* 2014; Petersen *et al.* 2014), individuals in a symptomatic stage of infection (Molassiotis *et al.* 2002; Chan *et al.* 2005), and a general population of HIV-infected men and women (Molassiotis *et al.* 2002; Adams *et al.* 2012*a*; Pence *et al.* 2014; Petersen *et al*. 2014; Yu *et al.* 2014). Four of the studies reported inclusion criteria based on depressive symptoms or disorder (Field & Kruger, 2008; Adams *et al.* 2012*a*; Pence *et al.* 2014; Petersen *et al.* 2014).

#### Study design

Five of the pilot studies randomized individual participants (Molassiotis *et al.* 2002; Chan *et al.* 2005; Bhana *et al.* 2014; Petersen *et al.* 2014; Pence *et al.* 2014) and one study randomized clusters (Nyamathi *et al.* 2012) to the intervention or control condition. Three studies were quasi-experimental, and did not randomize participants to condition (Jirapaet, 2000; Field & Kruger, 2008; Mundell *et al.* 2011). Three studies were pre-experimental with a one-group, pre-posttest design (Adams *et al.* 2012*a*, Pence *et al*. 2014; Yu *et al.* 2014). Most of the studies (8 of 12) measured effects at posttest only and utilized sample sizes appropriate for pilot studies.

#### Intervention content and delivery method

The interventions tested in these studies were diverse. Most interventions were designed for delivery within groups (Jirapaet, 2000; Molassiotis *et al.* 2002; Chan *et al.* 2005; Field & Kruger, 2008; Mundell *et al.* 2011; Ravaei *et al.* 2013; Bhana *et al.* 2014; Petersen *et al.* 2014; Yu *et al.* 2014), one was designed for delivery with individuals (Nyamathi *et al.* 2012, 2013), and two were pharmacological studies that utilized a clinic-based model for task-shifting antidepressant management (Adams *et al.* 2012*a*; Pence *et al.* 2014). The individual-level intervention (six sessions) addressed behavioral outcomes using psychoeducation, coping, and skills training (Nyamathi *et al.* 2012, 2013). Group interventions (1–12 sessions) targeted behavioral (Jirapaet, 2000; Molassiotis *et al.* 2002; Bhana *et al.* 2014) and psychological (Molassiotis *et al.* 2002; Chan *et al.* 2005; Field & Kruger, 2008; Mundell *et al.* 2011; Ravaei *et al.* 2013; Petersen *et al.* 2014; Yu *et al.* 2014) outcomes through utilization of art therapy (Field & Kruger, 2008), cognitive behavioral therapy (Molassiotis *et al.* 2002; Chan *et al.* 2005), peer support counseling/group therapy, coping and stress management (Molassiotis *et al.* 2002; Mundell *et al.* 2011; Yu *et al.* 2014), skills building (Jirapaet, 2000), and interpersonal therapy (Petersen *et al.* 2014). Both of the pharmacological interventions aimed to reduce depression symptoms over a period of 12 weeks through a task-shifting model (Adams *et al.* 2012*a*; Pence *et al.* 2014). A wide range of professionals and non-specialists delivered the interventions.

#### Control/comparison condition

Most studies (eight) used a comparison or control condition, with six of the 12 studies randomizing participants to condition (Molassiotis *et al.* 2002; Chan *et al.* 2005; Nyamathi *et al.* 2012, 2013; Ravaei *et al.* 2013; Bhana *et al.* 2014; Petersen *et al.* 2014). As shown in Table 2, these included, for example, standard of care, wait list control, and counseling as needed.

#### Outcome measures

All studies assessed mental health outcomes using various standardized psychological scales (Table 2). Depressive symptomatology was the most commonly assessed mental health outcome; one study (Pence *et al.* 2014) used major depressive disorder based on a symptom scale and physician assessment.

#### Preliminary intervention effects

Eleven of the 12 pilot, feasibility, or quasi-experimental studies demonstrated promising effects related to improvements in mental health (Jirapaet, 2000; Molassiotis *et al.* 2002; Chan *et al.* 2005; Field & Kruger, 2008; Adams *et al.* 2012*a*; Nyamathi *et al.* 2012, 2013; Ravaei *et al.* 2013; Pence *et al.* 2014; Petersen *et al.* 2014; Yu *et al.* 2014), or behavioral (Adams *et al.* 2012*a*; Bhana *et al.* 2014) outcomes. One study (Mundell *et al.* 2011) demonstrated mixed effects, with improvements in active coping and self-esteem in the intervention group, but not in depression or social support.

## Discussion

There is an urgent need to address mental health in the context of HIV/AIDS in LMICs, which bear the brunt of global HIV infections. Although previous reviews (Crepaz *et al.* 2008; Brown & Vanable, 2011; Clucas *et al*. 2011; Harding *et al.* 2011; Sherr *et al.* 2011; Seedat, 2012; Spies *et al.* 2013; Wu & Li, 2013) have identified a large number of RCTs to improve mental health in high-income settings, they have only included a small number of intervention trials conducted in LMICs. The purpose of this review was to systematically identify intervention trials that have addressed mental health among PLWH in LMIC settings, and to synthesize the lessons learned from those studies. Our review identified 22 unique intervention studies, 10 of which were evaluated using rigorous RCT methodology. Although this systematic search included a range of terms for mental health, only intervention trials with depression, anxiety or overall psychiatric distress outcomes were identified. Despite the fact that only a small number of full scale trials provided evidence for improvements in mental health, the findings of the review point to opportunities for further research on interventions to address the mental health needs of PLWH in LMIC settings.

The four RCT intervention trials in our review that demonstrated an impact on mental health primarily utilized a multi-component approach. Interestingly, the most robust outcomes, including over longer term follow up assessments, were found in community based trials (Li *et al.* 2010, 2011, 2012, 2014; Richter *et al*. 2014; Rotheram-Borus *et al*. 2014) that contextualized HIV/AIDS and mental health within family interactions or through peer support that addressed issues related to pregnancy and child outcomes. The inclusion of pilot studies and quasi-experimental trials in our review provided a framework for mental health interventions that are in the development phase and potentially proceeding to full scale trials. Although the study methods were acknowledged as less rigorous, many used RCT methodology albeit among small sample sizes, and all reported encouraging results using several innovative intervention approaches.

A number of studies, both RCTs and preliminary studies, were group-based interventions, utilizing a cognitive behavioral approach (including stress management and coping interventions), and often delivered in a task-shifting or task-sharing model with lay counselors or community health workers. Future directions explored in pilot trials included a measurement based care (MBC) stepped approach to antidepressant medication management (Trivedi *et al.* 2007; Adams *et al.* 2012*b*) that employed non-specialists to screen and monitor depressive symptoms, thereby supporting intervention at the clinic system level. These trials, as well as two others that evaluated psychotherapeutic approaches, were the only studies that used an inclusion criterion for mental disorder or depressive symptoms. Several of these pilot trials included an intervention focus or secondary outcome measure of adherence to ART. These study methods and intervention approaches point the way forward for mental health intervention trials with PLWH in LMICs, including integration with HIV care and treatment.

The studies we identified had methodological limitations similar to those documented in prior reviews of trials conducted primarily in HICs (Crepaz *et al.* 2008; Clucas *et al.* 2011; Sherr *et al.* 2011; Seedat, 2012; Spies *et al.* 2013). Of key importance only a few studies used mental disorder or above threshold symptom levels as an inclusion criterion for trial selection. This suggests that either the interventions may not have targeted PLWH experiencing significant psychological distress or that symptom levels were sub-threshold, making it difficult to demonstrate an intervention effect. In addition, a limited number of studies provided information on the cultural appropriateness of the intervention or the adaptation and validation of the mental health measures utilized (Bass *et al.* 2007). Several other secondary limitations of the methodologies used in our included studies should be noted. Even among the RCTs, the majority of the intervention trials assessed either immediate post- or short-term intervention effects, with longer term follow up assessments needed to determine sustainability of intervention effects. Most studies provided a general description of the intervention approach, but a detailed description of intervention components and fidelity to them in delivery would enhance the understanding of intervention outcome findings. Finally, intervention trials using non-specialists to deliver mental health interventions (i.e. a task-sharing model) did not provide adequate information on the training and supervision of the providers (Patel *et al.* 2007).

Despite the methodological limitations of the studies included in this review, it is encouraging to see an increasing number of mental health intervention trials for PLWH conducted in LMICs, which span a wide range of populations, countries, and intervention approaches. Additional lessons can be drawn from efficacious interventions for depression treatment in LMICs, (Bolton *et al.* 2003; Patel *et al.* 2007, 2009; Rahman *et al.* 2008), even if not specific to PLWH. Such interventions have been integrated into routine health care, adapted to local cultural context, and implemented by non-specialists. The intervention approaches for treating depression varied, but reflect approaches identified in this review for PLWH, including a collaborative stepped care approach (Patel *et al.* 2007, 2009), home-based individual CBT (Rahman *et al.* 2008), and interpersonal group therapy (Bolton *et al.* 2003). Although mental disorders in HIV care settings in LMICs often go undiagnosed due to lack of screening protocols (Breuer *et al.* 2014; Tsai, 2014), these studies suggest that integration of mental health screening and intervention into health care settings would be an effective approach for improving mental health among PLWH. Two of the preliminary studies we identified support the feasibility of a MBC approach for antidepressant medication in the HIV care setting (Adams *et al*. 2012*b*; Pence *et al.* 2014), similar to the stepped approach in the MANashanti Sudhar Shodh (MANAS) trial (Patel *et al.* 2007, 2009), yet more medication-based or combined medication and therapy based trials are needed.

CBIs, including problem solving, skills training, and stress management, commonly used in both HIC and LMIC, support the use of cognitive-behavioral treatments as a key mental health intervention approach. However, there is a need for cultural adaptation and tailoring mental health idioms to the local context, which may preclude the ready transplant of existing CBIs for PLWH. Research trials are still needed to test factors such as required intervention length for feasibility, effectiveness and maintenance of effect, and delivery by non-specialists. There is a need for task-sharing and interventions that can be delivered by non-specialists that are brief and scalable, while providing supervision and fidelity monitoring.

While mental health interventions for PLWH should draw upon the broader evidence of efficacious mental health interventions, they must also remain attuned to issues that are unique for a population living with HIV. These include potential barriers related to HIV-related stigma (Skinner & Mfecane, 2004), substance abuse co-morbidities (Gonzalez *et al.* 2011; Kader *et al.* 2012), and other disorders prevalent among PLWH such as PTSD (Machtinger *et al.* 2012). Multilevel system-strengthening approaches that integrate mental health care into HIV care and prevention within health care and community based organizations has been recommended (Joska & Sorsdahl, 2012; Lund *et al.* 2014). Areas for future research in LMICs include the integration of mental health treatment with adherence, HIV care engagement, and HIV prevention (Sikkema *et al.* 2010; Chibanda *et al.* 2014). Thus, one priority for future research is to conduct RCTs of mental health interventions that improve mental health and enhance HIV treatment and prevention.

The findings from this review of interventions to improve mental health among PLWH in LMICs also provide lessons learned and potential future directions to improve related efforts in HICs. Effective interventions in LMICs utilized family or multilevel interventions and were integrated within community based health care – approaches that contextualize mental health and provide an opportunity to address comorbidities. These approaches, in combination with addressing structural barriers to care such as poverty, health care access, and mental health care policies, are also relevant in HICs, especially in settings where health disparities clearly exist. Given the limited mental health resources in LMICs, including the absence of specialists trained in mental health treatment, the LMIC interventions incorporated approaches delivered by non-specialists. This task-sharing approach is also relevant and should be evaluated in HIC settings, particularly in communities and settings where access to mental health care is limited. Research methodologies related to monitoring intervention fidelity and supervision of non-specialists could enhance our understanding of key elements of effective interventions in both settings. Lastly, this review of interventions in LMIC settings offers lessons for the adaptation of available evidence-based interventions with attention to language, culture, and literacy, as well as feasibility of intervention length. These factors may also impact the effectiveness of mental health intervention for PLWH in HICs, and if better addressed, could improve the outcomes in higher income settings where disparities and cultural differences exist.

Our search strategy, while systematized, cannot guarantee the identification of all interventions to improve mental health among PLWH in LMICs, and omission of related intervention research is possible and may have influenced our conclusions. Unlike prior reviews (Clucas *et al.* 2011; Seedat, 2012; Spies *et al.* 2013), we intentionally cast a wide net that resulted in the inclusion of studies ranging from multilevel family and community based approaches to a group adherence intervention that also assessed the impact on depression. In addition, we included trials that reported mental health as secondary outcomes (4 of 22 studies). Although this approach broadly defined mental health and supports the importance of addressing mental health in context, a limited number of RCTs were identified, and only a portion of these trials provided evidence for the efficacy of the mental health intervention, even when looking for post-only mental health outcomes. Despite these potential shortcomings, this review provides an overview of the body of evidence available on mental health treatment of PLWH on LMICs, and offers suggestions for the path forward for understanding and addressing these needs.

### Conclusion

There is a paucity of empirical data investigating the effectiveness of interventions for mental disorders and psychological distress among PLWH in LMICs. The available data are restricted to several RCTs with widely varied approaches and methodology; and to several smaller pilot, and innovative studies. It is not possible therefore to describe from such a review, the nature, content, and delivery of an ideal intervention. Key issues such as adapting the intervention to suit local culture, language, and resource-limitations are typically not addressed. We are some way from being able to define an ideal intervention, and so a framework for building interventions of this kind is a desirable next step.
